# Supporting Mothers' Engagement in a Community-Based Methadone Treatment Program

**DOI:** 10.1155/2013/987463

**Published:** 2013-05-07

**Authors:** Nicole Letourneau, Mary Ann Campbell, Jennifer Woodland, Jennifer Colpitts

**Affiliations:** ^1^Faculty of Nursing, University of Calgary, 2500 University Drive NW, AB, Canada T2N 1N4; ^2^Department of Psychology, University of New Brunswick, Saint John Campus, P.O. Box 5050, Saint John, NB, Canada E2L 4L5; ^3^Faculty of Nursing, University of New Brunswick, P.O. Box 4400, Fredericton, NB, Canada E3B 5A3

## Abstract

Unmanaged maternal opioid addiction poses health and social risks to both mothers and children in their care. Methadone maintenance treatment (MMT) is a targeted public health service to which nurses and other allied health professionals may refer these high risk families for support. Mothers participating in MMT to manage their addiction and their service providers were interviewed to identify resources to maximize mothers' engagement in treatment and enhance mothers' parenting capacity. Twelve mothers and six service providers were recruited from an outpatient Atlantic Canadian methadone treatment program. Two major barriers to engagement in MMT were identified by both mothers and service providers including (1) the lack of available and consistent childcare while mothers attended outpatient programs and (2) challenges with transportation to the treatment facility. All participants noted the potential benefits of adding supportive resources for the children of mothers involved in MMT and for mothers to learn how to communicate more effectively with their children and rebuild damaged mother-child relationships. The public health benefits of integrating parent-child ancillary supports into MMT for mothers are discussed.

## 1. Introduction

In North America, opioid addiction to methadone, hydrocodone, and oxycodone has been described as an epidemic [1, 2]. In Canada, nearly 10% of women self-report an addiction to some form of illicit substance (e.g., cannabis; [3]) and nearly 2% abuse highly addictive opioid substances like oxycontin and heroin [4]. Many of these women are mothers of childbearing age [5] and face greater challenges than nonusers with raising their children and meeting their children's developmental, social, cognitive, and emotional needs [6]. Substance abusing mothers are at increased risk of developing affective disorders, low self-esteem, anxiety, and depression and are often challenged by social isolation, reduced support networks, and exposure to violent relationships [7] and all of these factors can influence a mother's capacity to parent her children [8, 9]. Taken together, unmitigated maternal substance abuse exacts social and health care costs by increasing the need for child protective, mental health and criminal justice services.

Protecting children from exposure to maternal substance abuse is a public health priority [10, 11], particularly for nurses who work with childbearing families in the community [12]. Children exposed to maternal substance abuse are at increased risk for developmental problems, such as cognitive deficits, language delays, emotional problems, behavioural disorders, and becoming substance abusers themselves [13, 14]. The increased likelihood of intergenerational transmission of substance abuse is linked to addicted mothers' parenting behaviour, often characterized as neglectful [6]. Notably, substance abuse is one of the top three stressors influencing children's development, along with mental illness and family violence [15], and the presence of maternal substance abuse is a greater risk factor to children's development than paternal substance abuse [13]. Indeed, the American Academy of Pediatrics recently described maternal addictions as toxic to children's development and called for preventative public health interventions to reduce the detrimental effect of maternal addictions [16]. Given the increased risk for a variety of adverse health outcomes, opioid dependence is a growing public health concern [17], particularly for mothers responsible for the care of children [18]. Nurses engaged in public health practice are at the forefront of ensuring these families receive appropriate support [12].

One public health approach that nurses use to support and refer patients to manage opioid addiction is methadone maintenance treatment (MMT) [12]. Compared to control groups and other forms of opioid addiction intervention, MMT is an effective public health strategy. [19–21]. MMT has been associated with reduced opioid use [22–25], improved health outcomes and reduced mortality rates [26–30], enhanced mental health status [31, 32], and improved social and occupational functioning [26]. In some cases MMT has led to reductions in criminal activity [23, 33] and nonopioid drug use [22], but these have not been consistent findings across studies [34, 35]. Furthermore, involvement in MMT has been found to positively influence retention in addiction-focused treatment [23]. Amato et al. [36] conducted a meta-analysis of 52 studies on MMT and other forms of substance abuse intervention for opiate addiction and found that participants of MMT were less likely to prematurely leave treatment compared to clients who were receiving no addiction services, methadone detoxification, or buprenorphine maintenance.

Although MMT appears to have meaningful benefits, less is known about mothers' experiences and how to promote and maintain mothers' engagement in treatment. Given the impact of parental addiction on children, engagement in MMT may create opportunities for enhancing mothers' parenting capacity through its positive impact on mothers' emotional, behavioural, and physical health well-being. However, little knowledge exists about the influences of MMT on mother's perceptions of their parenting or on the types of parenting supports and resources women need to facilitate their long-term engagement in MMT. Insights from the field have identified numerous barriers for mothers that influence their decision or capacity to seek and enter substance abuse treatment, including social stigma [37], lack of gender-specific treatment to address women's psychosocial needs [38], fear of losing custody of their children, lack of childcare during treatment, fear of partner retaliation or violence [22, 28, 39], and living with an opioid-dependent partner [40]. Nonetheless, for some mothers, being a parent may be a powerful incentive to seek professional help in addressing their addiction [16, 29].

While MMT has the potential to enhance both a mother's individual functioning and, thereby, her parenting capacity, mothers also experience challenges associated with being a parent that interfere with their engagement in MMT [41]. Research from the Center for Substance Abuse Research [41] has suggested that programs which bring together women and their children may be more effective at retaining women in treatment and have demonstrated long-term effects. This conclusion has been reaffirmed in two recent systematic reviews by Niccols and colleagues [42, 43], who found that substance abuse programs with integrated components focused on addiction, parenting, and child-related services lead to positive outcomes for both mothers and their children across a number of domains (e.g., parenting skills and child development). To date, however, researchers have not explored mothers' perspectives of the supports and resources needed to assist them with their parenting or to successfully engage in long-term MMT. Neither have researchers explored the perspectives of expert service providers. Consequently, the current research was designed to fill these gaps by accessing both experiential (mothers) and expert (service providers) knowledge of methods and means to promote mother engagement in MMT and to seek their perspectives on the characteristics of the desired parenting supports and resources. Specifically, the primary goal of the study was to explore the experience of mothers participating in an outpatient Atlantic Canadian MMT program and their service providers to gain an appreciation of the impact of this intervention on parenting and mother-child relationships. The secondary goal was to learn from mothers about the types of supports and services that might assist them with their mother-child relationships and parenting to maintain engagement in MMT.

## 2. Methods

The Atlantic Canadian MMT program, which began operation in 2005, is staffed by a multidisciplinary team of nurses, physicians, and social workers who advocate a harm reduction model of care. Referrals to the program come from a variety of sources including public health nurses, primary care physicians, psychologists, detoxification centre staff (physicians, nurses, or psychologists), or self-referrals. The program provides comprehensive, client-centred, community-based services to its participants in the form of access to prescribed methadone, individual counseling and group counseling, and educational programs associated with addiction, as well as facilitation of referrals to other relevant community resources as needed, such as mental health services and social services to assist with housing, financial, and parenting supports. The program does not actually offer anything specific for mothers and their children. The model of intervention is based on Health Canada's 2002 [5] guidelines for the best practices in the delivery of MMT interventions. Participants are eligible for the program if they have an opioid addiction but can be polysubstance abusers. Individual and group-based interventions focus on themes of addiction, self-esteem, infectious disease, hope, wellness, and anxiety, as well as support for new mothers or expecting mothers. Long-term retention in this program is a desired outcome given the maintenance focus of treatment once a client has stabilized on methadone. A recent evaluation of the program reviewed all cases (*n* = 458) admitted to this program between 2005 and 2010 [33]. The greatest gains were achieved during the first year of treatment, with self-reported improvements in opioid drug use, employment status, health, and mental wellness, as well as reduced involvement in criminal activity, including drug-related criminal activity.

After ethical approval was obtained, the study was performed in accordance with the ethical standards established in the 1964 Declaration of Helsinki and the 2010 Canadian Tri-Council guidelines for ethical research. All participants provided informed consent prior to study participation. Data collection occurred over a 12-month period between 2010 and 2011.

### 2.1. Participants

Mothers in outpatient treatment at the MMT program were recruited to participate if they met inclusion criteria. Eligible mothers ranged in age from 16 to 55 years and must have been actively parenting children between birth and 18 years of age. No specific duration limit was set for involvement with the program, but all cases were past the 3-month stabilization phase of the program. A combination of convenience, snowball, and word of mouth strategies were used to recruit the sample. Advertisements inviting mothers to participate were placed in strategic locations at the addiction treatment centre and presentations describing the study were made, individually, to mothers attending MMT. Mothers received a $30 grocery certificate to thank them for their participation in the study. Of approximately 90 mothers enrolled in MMT, [33], 12 mothers volunteered to participate, providing sufficient numbers for qualitative data saturation (when no new themes emerged in the data) [44].  Table 1 contains a more detailed summary of the demographic characteristics of these 12 participants. Addiction service providers working with the Atlantic Canadian MMT program were recruited through consultation with the research project's advisory committee that was formed specifically for this project and was composed of agency administrators, service providers (nurses and social workers), and a client representative from the treatment centre. A total of six service providers were recruited out of a potential pool of nine, including four social workers and two ancillary support providers who had administrative relationships with program clients (program coordination).

### 2.2. Semistructured Interviews

Mothers and service providers were interviewed using semistructured interview guides developed specifically for the study. These interviews were designed to determine their perspectives on parenting resources and supports to engage in long-term MMT. Although the interviews were structured slightly differently for mothers and service providers, the content tapped by the questions was consistent between the two interview protocols. Specifically, mothers and service providers were asked about (1) parenting support needs, (2) barriers to support faced by mothers, (3) knowledge and use of support resources, and (4) preferences for parenting support and resources to facilitate engagement in community-based addictions treatment.

### 2.3. Procedure

All service provider interviews and most of the mother interviews were conducted at the addictions treatment centre. One of two female graduate students conducted the interviews after receiving training in interviewing skills. Childcare was provided for the children of mothers as necessary. One mother interview was conducted in a private room in a public library closer to her home. All interviews were audio recorded for later transcription and analysis using NVivo version 9 software. Interviews were transcribed verbatim and subjected to a validation check for accuracy. Demographic information (age, gender, and ethnicity) and family composition (number of children and marital status) were collected prior to the interview.

Thematic content analysis was employed to examine the data, consisting of several steps [45]. First, a category system (coding framework) of key themes was inductively created by the first two authors and an academic colleague to capture all data from a subsample of randomly chosen interviews. Second, two trained research assistants read and coded all interviews under the guidance of the first two authors. Additional codes were added to the coding framework as new themes emerged from the data. After all data were coded, identified overarching themes and subthemes were identified that addressed the research questions. The final step of the analysis involved triangulating data by source (mothers in MMT/service providers) to enhance the value and validity of the study's findings.

## 3. Results

### 3.1. Trajectory of Addiction and Treatment: Addiction Onset, Reasons for Seeking Treatment, and Perceptions of MMT Impact

The mothers described the onset of their addiction to opiates and other substances as occurring through a variety of circumstances. The most common circumstance was “from a medical issue,” for example, chronic back pain. Others noted that they became addicted after being introduced to drugs through a boyfriend or partner, experimentation, or because drug use seemed glamorous. A third cited their children as the main impetus for seeking treatment. Mothers wanted to be better parents:

I decided “you know what, it's getting out of control.” (…) And not only that, my daughter was twelve going on thirteen and I thought “you know what? I do not want her growing up in a city either and maybe she'll turn out like I am right now (…). (MMT 04)

Similarly, a number of service providers attributed mothers' motivation to address their drug use to their desire to be a better parent. As one service provider suggested, *“that's number one, try and get cleaned up to be better moms, to be back in their (children's) lives where they want to be.”* (SP 01)

When questioned about how MMT affected them as a person, most mothers thought it did so in a positive way. After joining the MMT program, they felt better or stronger than before and were successful in getting and staying clean, and some thought the program saved their lives. According to one mother,

It [MMT] has made me better; (…) so much that I do not want drugs, I do not think about them. I'm past it, I am. The only time I think about drugs is when I'm here [at treatment]. (MMT 04)

### 3.2. MMT, Parenting, and Parent-Child Relationships

When asked about the effect of MMT on mothers' ability to parent, service providers described a host of improvements including making mothers more attentive to their children's needs, teaching mothers useful life skills and parenting strategies, and providing structure and stability, as well as making mothers' lives safer. One service provider commented,

They learn a lot of really good skills related to their drug use and related to their recovery (…). They learn about relationships (…). They learn about their triggers and their cravings and what sets them off and makes them relapse. (SP 01)

When asked how the MMT program had affected their parenting, many women felt it had made them better mothers. They felt more attentive to their children's needs and better able to engage with them, as illustrated in the following statement:

I'm not foggy anymore, I'm more clear and I'm just a better mother. I play with [my children] all of the time, I do activities, I read to them, whereas before I just used to feel like everything I did was a chore. But now I just take everything and I'm grateful for it. (MMT 03)

Most claimed their relationship with their children had improved after attending the MMT program. Mothers reported that they had more time and energy to devote to their children and were more emotionally available. Their relationships with their children were also described as more open, honest, and marked by a stronger bond. Overcoming their addiction allowed some to get their relationships with their children “back to normal” after a period of relative neglect:

Before you are on Methadone it just feels like you are lying all of the time, lying to yourself. When I was on the pills I wasn't active and wanting to do stuff like I should have, like going to school functions and stuff like that. The father would be doing all of that. (But) now that I'm on Methadone I feel better and I can do all of that stuff. (MMT 02)

Many mothers felt that the MMT program had a positive impact on their relationships with their families. Regarding their children's personal development, however, the vast majority thought MMT had no significant impact at all. While describing both positive and negative changes in their children's behaviour, mothers attributed these changes more to the removal of drugs in their lives rather than the MMT program itself. Some reported that their children were happier, while some children struggled with the frustration of having to attend sessions and take daily trips to the pharmacy with their mothers.

### 3.3. Support Needs and Preferences

When discussing which of their support needs were successfully met by MMT, mothers indicated that the program was helpful by providing them someone with whom they could talk about their issues, access to classes on parenting skills, readily available social workers or counselors, and emotional support. According to one mother,

Just having somebody to talk to and being able to come in and give you different resources too, because I really did not have a clue, I did not ever think I would have kids. It was just all thrown on me so it was nice having that support. (MMT 12)

While the methadone treatment facility is not designed specifically for mothers, it does offer access to external programs that address their unique needs, such as parenting groups and classes on parenting skills. However, the MMT treatment group forum offers an opportunity for mothers in a similar situation to commiserate with each other about parenting concerns. As one service provider stated,

When moms learn from some of the other participants in a group about what they've gone through, it might be a parenting issue, that's where a lot of the moms get some of their support from peers, is, in groups or people who have gone through that before, and, they say “My kids are just back in my care and I'm having a real hard time saying no and maintaining boundaries. Has it happened with anyone else?” And then moms receive feedback that way. (SP 01)

#### 3.3.1. Barriers to Support

Mothers discussed the support needs that were not met by MMT. The majority of complaints pertained to the absence of programs within the context of the MMT program itself that addressed the issue of children, such as services facilitating the bond between mothers and children. As one mother suggested,

They should have a program [at MMT] to watch your kids or anything while you go to group and stuff (…). We've even said it to them but they said they do not have [a service] to watch kids over there. (MMT 05)

They also encountered a number of barriers to support in their daily lives and in their attempts to access the MMT program. The two most common barriers were a lack of daycare facilities and convenient, affordable transportation. Transportation issues generally involved the complicated logistics of making regular trips to the centre, as few mothers had access to their own vehicles. According to one mother,

My issue was when I first go in the Program I was attending a meeting that I really enjoyed and we did not have a car at that point and they were giving us transportation to the meeting and then all of a sudden it stopped and I'm like “well how am I supposed to get there?” (…) I think there should be transportation for people that need it. (MMT 07)

Compounding this problem was the distance between clients' homes and the MMT centre, which some felt was too out of the way and was particularly difficult to use with children in tow. One mother described her frustration with taking the bus to treatment, stating,

“It's hard to get transportation out here sometimes. When I first lived here, I was taking a bus with a newborn baby and [my oldest child], with a four year old on a great big double stroller by myself (…). (MMT 05)

The cost of transportation was another prohibitive factor for some mothers, as cab fare and the cost of taking a bus surpassed their budget. As one mother commented,

“Having to come into town everyday was like a five dollar trip because coming in and going back out again, so that caused barriers. (MMT 06)

They also lacked the financial means necessary to enroll their children in daycare, which limited their ability to attend sessions and was a significant barrier to their recovery. As one mother suggested,

Too bad they did not have childcare here at [MMT] and then I think more people would come to the meetings because it would be easy for them. It is hard finding sitters, (…) what do they do? (MMT 07)

Wait time was an additional barrier to treatment for some, as were more personal issues, such as: lack of family support and embarrassment or fear. Another barrier identified by mothers was a lack of information about the availability of MMT, both within addictions services and the greater community. One mother commented,

“I just think its lack of knowing about it. People do not know. (…). Hopefully there is for people who need it. (MMT 07)

#### 3.3.2. Family and Community Supports

Of all the potential support resources available outside of MMT, mothers' parents were called upon most often and adopted the widest variety of supportive roles. Their parents provided basic needs, such as childcare, emotional support, and affirmational support. In contrast, some mothers revealed that they were reluctant to leave their children with their own parents, due to troubled interpersonal relationship histories. However, their parents were still a better option than other members of their social network, who were often drug abusers. As the following passage illustrates, they often need their parents to care for their children during recovery,

…I was sick, like really, really sick because I couldn't get my methadone. So I called my mom and I knew what was going to happen. I knew the outcome of it and I told her and I said, “Please come get [child].” It broke my heart to do that but I told her, I said “Come and get him because I cannot sit here and be sick and take care of him and I know what I have to do to not be sick.” (MMT 05)

Mothers' friends and siblings also helped by providing childcare. One spoke about being able to talk to a friend about her urges to use,

If she's having a bad day or I'm having a bad day and if she wants to use, she'll tell me instead of going out and using. It's just better to talk about it (…) than keeping it in because if you keep it all in and you end up just going and doing it then. (MMT 05)

Mothers also found support within the wider community, from counselors and social workers. One mother stated,

There's a place over north though that gives you diapers and formula and stuff. (…) I'm on social assistance so they give you lots of help. Bus passes and things like that to help you get back and forth. (MMT 12)

#### 3.3.3. Preferred Medium for Support Delivery

All twelve mothers were universally in favour of one-on-one support compared to group support. According to service providers, one-on-one support allowed providers to focus on the mother, addressing her needs specifically, rather than in general terms. The private nature of sessions allowed mothers to share intimate details they might not be comfortable discussing in front of a group. One service provider commented,

Especially the moms that have the kids, a lot of them have taken advantage of individual counselling, the one-on-one counselling, because lots of times their issues are so personal that do not really want to air them in a group. (SP 04)

Group support, however, was also widely suggested by mothers, as it allowed them to support one another,

…they cover things in groups that you might not know how to deal with, like people give their advice, if you are craving what do you do? You might not think of all of these ideas by yourself but you get a lot of them at groups and just other people's experiences because they've been in the Program longer… (MMT 07)

#### 3.3.4. Preferred Method of Contact with MMT Service Providers

For preferred method of contact, all twelve mothers favoured face-to-face interactions with service providers. This was followed by telephone contact, which was favoured in part for its ability to provide instant, twenty-four hour access to service providers. As one stated *“it would be nice for people to know that they could pick up the phone and have that instant kind of release and advice.” (MMT 11)*

Service providers recommended face-to-face, telephone, and online treatment. Service providers preferred face-to-face contact given its ability to facilitate intimate communication. A service provider stated,

I prefer face-to-face, because that way you can gauge peoples cues, you can tell if someone is uncomfortable, you can tell if someone is really engaged, and it gives you just that opportunity to connect. (…) when you sit with someone face to face and they're letting you in on what's going on and sharing that with you there's an inherent trust there that's very different than over the phone. (SP 01)

Telephone support was considered an important resource, as it allowed mothers with hectic schedules to contact support staff should they have a crisis or need specific information. Some service providers reported that telephone support worked best as a secondary option. As one provider suggested,

I do not think telephone support exclusively, but I think telephone support is important. For example, we have moms that telephone support is huge, they can call and do call often and we are available so that we can talk to them (…). We cannot conduct long-term, significant therapeutic counselling on the phone, but we certainly can provide the affirmations, the support, the sort of assurances, the reassurances, the addressing the immediate perception or an immediate crisis, all of those sorts of things. (SP 03)

Like telephone support, computer-based support was seen as a useful secondary resource, particularly for providing a convenient access point for information about addiction recovery. However, these service providers expressed concern about mothers' ability to access computers. A service provider commented,

I think moms that have a hard time getting out, that might be a good way for them to be able to do that because they could go online. [My concern is] do they have computers? (SP 05)

#### 3.3.5. Preferred Facilitator for Support Provision

When discussing their preferred support provider for support groups, most mothers suggested a peer—a former addict who had successfully gone through treatment. They felt more comfortable talking to someone who knew what they were going through firsthand. As one mother commented,

Because no one really understands unless you've been through it yourself, really, they do not. I mean they try to and God bless them but they do not, so I think that is the best person to talk to or someone who at least understands addiction. (MMT 07)

Some thought a professional facilitator would be the best, while others found a combination of the two to be ideal. In contrast, all of the service providers felt that support groups should be facilitated by professionals, because of their specialized education and training, and some discussed the possibility of a combination of professional and peer facilitation.

#### 3.3.6. Preferred Location of Support Provision and Support Topics

For the ideal location, some mothers thought the program's present community location was fine. Others thought it would have helped to have sessions closer to their homes, or else somewhere more generally accessible. One mother suggested,

It would have to be somewhere where everyone can get to, like maybe somewhere central. Somewhere it's easy to get to, accessible. (…) I think if it is just a walk-in thing it would work better because when you go in there on your terms. (MMT 07)

When asked what topics the ideal treatment would cover, mothers suggested parenting and affirmational and emotional support. According to one mother,

Things are going to change in the household when someone starts getting well. Changes the whole dynamics of the household because when you've got somebody who's using (…) the child becomes the parent. That whole dynamic is going to change. All of a sudden, the child whose been probably getting away blue murder because he's the parent is suddenly going to be the child again… (MMT 06)

For the ideal duration of MMT programs, mothers typically suggested between an hour and an hour and a half. For ideal frequency, mothers opted for once or twice a week.

#### 3.3.7. Desired Support for Mothers

Lastly, mothers were asked to list potential areas of support they would find useful in an ideal MMT program. The most common suggestion was emotional support. An extension of this, group support, was also suggested.

Some people do not have family, some people do not have friends so it would be nice to have somewhere where they could go and talk to people if they needed to or even a place where moms could get together, (…) I think a lot of parents get discouraged too once they lose their kids they think there is no hope. (MMT 07)

In addition to emotional support, mothers felt information about methadone treatment would be important. One suggested a fairly extensive program for children whose parents are undergoing treatment, teaching them about methadone and addiction as well as educating parents on practical aspects of parenthood, such as how to enroll children in school. Another suggested,

When it comes to the children, I think they need education as well… (For example) that the methadone is not another drug, that it is a process of helping to get well, because there's a lot of information out there where people are saying, “Oh they're just going from one drug to another” and then the kids get fed up. (MMT 06)

Two service providers also believed that treatment should extend beyond mothers and address the needs of children as well. It is important, they felt, to treat children as individuals in need of counselling instead of roadblocks to their mothers' recovery. Furthermore, while mothers and children must both be addressed in treatment, so too must the bond between them. Facilitating stronger bonds between mother and child will help the family as a whole. One service provider commented,

What's going on with women's relationships with their children? With themselves? Are women's needs being met? Because when women's needs are being met, they're better able to meet the needs of their children. (SP 01)

## 4. Discussion

MMT has been shown to be a useful treatment for opioid addiction (e.g., Johansson et al. [23]) in targeted public health services. By participating in substance abuse treatment programs like MMT, mothers struggling with addiction not only stand to help themselves but also may have the opportunity to minimize the risk of poor developmental, mental health, and behaviour outcomes for their children [43], reduce the likelihood of intergenerational transmission of addiction [18], and limit the social and health care costs associated with addiction. Extensive research has addressed the negative effect of substance abuse on mother-child relationships and child development (e.g., Bowie [39]), but very little research has examined mothering in the context of treatment for opioid addiction and general substance abuse. Existing research has suggested that motherhood status [40], residing with one's children [46], and enhanced social services [40] can each influence treatment success, as can integrating parent and child-focused interventions within substance abuse treatment for mothers [42]. However, mothers who decide to seek treatment still face significant challenges that can then interfere or complicate their engagement in substance abuse treatment, such as poverty, prior experiences of losing custody/access to one's children, inadequate/unstable housing, parental stress, domestic violence victimization, and insufficient or absence social support [47]. Thus, the current research examined mothers' and service providers' perspectives on their experience of being mothers in MMT, and on the parenting supports and resources that would assist mothers in successfully engaging in long-term MMT. The information gained from the current research will be of value to public health nurses who deliver substance abuse treatment services, as well as to researchers, managers, and policy experts who develop best practice models for such public health interventions.

A significant strength of the study was that the perspectives of mothers and their service providers were largely in agreement [48]. Many of the women in the current study chose MMT because they were often motivated by the desire to be better parents. One of the major barriers for mothers seeking and/or maintaining treatment for substance abuse is the fear of losing custody of their children once professionals become aware of their addiction issues [38, 49]. Similarly, Hughes et al. [50] found that mothers who were able to retain care of their children while being engaged in residential treatment programs have significantly better outcomes than mothers who did not retain their children. Positive outcomes also have been found for mothers who attended special mother-child service as part of their addiction intervention [51]. In both of these latter two studies, retention and successful discharge from treatment were enhanced by the presence of children in the residential care settings. In light of these types of findings, child-centered policies have been criticized for focusing more on the child and limiting maternal rights rather than promoting mothers' health, safely, and capacity to parent their children [52]. However, research into the role of children in mothers' treatment success has been mixed. For opioid-dependent women, having a higher number of children has been associated with poorer treatment history; that is, parenting responsibilities can limit treatment-seeking behaviours [40]. Faupel and Hanke [53] also noted a significant negative relationship between motherhood and enrolment in community-based treatment. Thus, greater awareness of these barriers and how to overcome them within the context of a community-based substance abuse treatment program are required.

Findings from this study agree with others' observation that while MMT engagement leads to more positive outcomes for mothers, accessible and safe childcare and family responsibilities act as barriers to MMT even when this service is available within the community they reside [49]. Compounding the childcare issue can be the impediment of transportation. For the mothers in the current study, attending treatment meant traveling long distances, often by means of public transportation that involved multiple bus transfers and reliance on third parties to provide a drive to the treatment site. Although not specific to mothers or women, Burbridge [33] reported that 42% of male and female clients in the same MMT program from which our mothers were drawn resided between 100 and 200 km away from the treatment centre and another 2% lived more than 200 km away. Thus, providing onsite childcare and easier access to efficient transportation to attend MMT services would make treatment much more accessible for mothers. These are public health issues that nurses engaged in MMT can address through patient advocacy and policy influence.

Mothers' perspectives in the current study echo those of Lundgren and colleagues [54] who advocated for childcare services for mothers in MMT and the need to develop specific parenting support services to promote engagement in MMT, such as skills training and parental counseling. These services may be provided in typical primary health care settings by public health nurses. The mothers in the current study specifically described a need for interventions that could help them rebuild relationships with their children that have been damaged or strained by the addiction. The provision of parent and family-based interventions has been shown to reduce parental stress in parents engaged in MMT, while also reducing problem behaviour in their children and enhancing their family functioning [10, 11, 42, 43]. Thus, multicomponent programs that address addiction issues, family dysfunction, and parenting have the potential to maximize treatment gains for both mothers and their children. The need for this type of intervention is not unique to mothers in MMT, as experts have also noted a similar need for fathers [55]. Further research is needed to establish ways that addiction services might address these parenting capacity needs through the provision of childcare, parenting skills, and family-based interventions. Given public health nurses' frequent focus on childbearing families, better integration of services for mothers receiving MMT into the existing public health service network is an area worthy of exploration.

Previous research has revealed that limited supportive treatment services are available for mothers who abuse substances relative to what is available for men and single women [38]. Mothers in the current study revealed that they relied most heavily on their family and friends for support during treatment, followed by other community and service providers. According to the mothers in the current study, MMT programs should focus on providing greater emotional support and encouragement to mothers, provide information to mothers about how to speak with their children about addiction and the recovery process or offer programs for children to attend on these topics, and provide services to better promote parenting skills. In general, mothers believed that the formal adoption of these parent-child focused components to their MMT program would enable them to strengthen their relationships with their children and enhance their parenting capacity. The only program available directly through this particular MMT service that pertained to children was geared toward expectant and new mothers. Although these maternity focused interventions with mothers dependent on substances have been shown to have value for reducing risks to both the mother and her infant [56], they do not meet the needs of mothers who are raising older children and adolescents. Formal partnerships with community and public health programs and social service agencies that provide parenting enhancement focused interventions for mothers in substance abuse treatment appears to be a valuable component of addiction intervention for mothers, for both them and their children. Other addiction programs have recognized the value of going beyond treating the substance abuse by means of multidimensional need screenings, cross-agency case planning, and service partnerships, such as that used by the Breaking the Cycle program in the United States for criminal offenders [57] and comprehensive outreach support services characteristic of such programs as the Sheway Project in Canada, which assists high-risk women who are pregnant or caring for infants [47]. The Sheway Project has adopted a harm reduction approach to its work with mothers and emphasized a nonjudgmental and respectful decorum towards mothers amongst its staff and service delivery to promote engagement. Evaluations of the Breaking the Cycle and Sheway Project programs have demonstrated improvements in participants' family functioning [47, 57]. Poole further noted benefits of the Sheway Project with regard to stabilizing the housing situation of mothers, enhancing mother's engagement in pre- and postnatal care, and the likelihood of mothers retaining custody of their children. However, Poole's evaluation identified gaps with regard to services for the partners of the mother's accessing addictions treatment and their children who were beyond the target age for the program of 18 months of age, parenting services, and other supports to provide safety and security to these families.

In the current study, the service providers who participated in our interviews appeared to appreciate mothers' experiences and recognized the complexity of being a parent in treatment. However, this is not always the case. Previous research has found that service providers sometimes hold stigma-related beliefs, stereotypes, and negative attitudes about mothers with addiction issues, and concerns for children of ten supercede concerns for mothers. These views can translate into judgments and misrepresentation of mothers by staff, which then interferes with a mother's willingness to engage in addiction treatment and support services for her children [58]. A qualitative analysis of service providers who provided health services to antenatal mothers with addiction issues revealed that staff tended to be more accepting and empathetic towards mothers with an addiction issues when these staff had a better understanding of the experience, challenges, and complexities of substance-dependent mothers [56]. Thus, staff working with substance-abusing mothers and their families are likely in a better position to overcome barriers to patient engagement when they can use their empathy and understanding to realistically support and encourage mothers, rather than to judge or hold mothers to negative expectations.

In addition to parent-child focused supports, mothers in the current study also spoke about the nature of service delivery. Many felt that the provision of support through individual counseling would give them an opportunity to explore personal issues in a confidential setting that goes beyond the direct addiction issues. These mothers also expressed value in group sessions as they viewed this medium of intervention as being able to provide them with the emotional support and words of encouragement they desired through the sharing of similar stories and experiences. While face-to-face support was by far the preferred mode of contact for these mothers with their service providers, they also recommended greater use of telephone contact because it would allow for 24-hour access to service provider support. In addition, many mothers wanted MMT to incorporate supportive resources that were facilitated by a peer who has successfully gone through treatment.

### 4.1. Study Limitations

First, the sample size for both mothers and service providers was low. Although data saturation was obtained for the mothers' data, this was not ensured in the service providers' data. Nonetheless, the themes identified in the 12 mothers' and six service providers' interviews shared a number of commonalities that lend credence. Thus, the current data likely reflects the experience of some women and service providers involved in this particular MMT program but may not necessarily fully generalize to the experience of mothers who chose not to participate or to mothers in other MMT programs. Fortunately, almost all of the staff employed with this MMT program volunteered to participate in the study. A second major limitation of the current research was the lack of voice given to the children of the interviewed mothers. Inclusion of age-appropriate child-focused interviews and assessment methods would have allowed the research findings to be further contextualized from the perspective of children in terms of their needs for supports and services while their mothers are engaged in treatment.

## 5. Conclusion

Mothers who struggle with addiction face significant barriers to engaging in substance abuse treatment, especially when treatment requires long-term commitments like MMT. In public health, MMT is the gold standard for helping individuals overcome addictions to opiates, but these interventions must be delivered in a manner that is sensitive to the needs of mothers and with an appreciation of the potential impact such programs have on parenting capacity and promotion of the well-being on the children of these clients. Inclusion of child and family-focused interventions into substance abuse treatment models that provide service to mothers is essential and indeed recommended. Mothers face great barriers to remaining engaged in community-based MMT due to challenges accessing safe and reliable childcare and accessing reasonable and reliable transportation to and from treatment sites. Public health nurses and other treatment providers and program managers must deliberately and creatively present solutions to these barriers to maximize the engagement of mothers in treatment. However, it is important to acknowledge that research often falls behind innovative clinical practice, and many MMT programs in public health are likely already working to address these solutions.

## Figures and Tables

**Table 1:** Demographic profile of participating mothers.

Demographic variables	*n *	*M* (SD)
Age		30.75 years (7.42)
20–24 years	2	
25–29 years	3	
30–39 years	3	
40+ years	2	
Current employment	
Work part time	1	
Homemaker	5	
Unemployed	5	
Education		
Partial high school	8	
Completed high school	2	
Partial technical school	1	
Completed technical school	1	
Ethnicity		
Caucasian	12	
Other	0	
First language		
English	10	
Other	0	
Disability		
Yes	3	
No	7	
Marital status		
Legally separated	2	
Boyfriend/girlfriend	4	
Ex-boyfriend/girlfriend	3	
Widowed	1	
Number of children		2.45 (1.44)
1	3	
2	4	
3	2	
5	2	
Children's age		70.6 months (78.19)
Social assistance		
Yes	9	
No	0	

Note: some participants did not provide responses to each of the demographic questions. Thus, totals do not always sum to 12 participants, but range from 9 to 12.
